# Antimicrobial susceptibility testing and reporting practices of public hospital microbiology laboratories in Greece, 2022: A national observational survey and call for action

**DOI:** 10.2807/1560-7917.ES.2023.28.34.2200766

**Published:** 2023-08-24

**Authors:** Sofia Karagiannidou, Ioannis Kopsidas, Michalis Polemis, Kyriaki Tryfinopoulou, Theoklis Zaoutis

**Affiliations:** 1 National Public Health Organization (NPHO), Athens, Greece; 2 Center for Clinical Epidemiology and Outcomes Research (CLEO), Non-Profit Civil Partnership, Athens, Greece; 3 Central Public Health Laboratory, National Public Health Organization (NPHO), Vari, Greece; 4 The 2nd Department of Pediatrics, National and Kapodistrian University of Athens, ‘P. & A. Kyriakou’ Children's Hospital, Athens, Greece

**Keywords:** Antimicrobial Susceptibility Testing, Practices, Capacity, EUCAST, CLSI, Microbiology labs

## Abstract

Antimicrobial resistance (AMR) in Greece is among the highest across the European Union/European Economic Area (EU/EEA), with high AMR rates even to last-line antibiotics. To better understand the clinical microbiology laboratory practices and capacities in species identification and antimicrobial susceptibility testing (AST) across public healthcare establishments in Greece, we sent a questionnaire to 98 of 128 public hospital microbiology laboratories between 1 February and 1 April 2022. Of the 73.5% (72/98) that responded, 51.4% (37/72) reported using EUCAST guidelines. Two of three laboratories used an automated instrument for species identification and AST for all laboratory samples. Broth microdilution for colistin susceptibility testing was used by 46 of the laboratories, more frequently in larger (> 400 beds) versus smaller (< 400 beds) hospitals (90.5% (19/21) vs 52.9% (27/51) respectively, p = 0.011). MALDI-TOF mass spectrometry was available in one of 10 laboratories, and more often in larger compared to smaller hospitals (p = 0.035). Although the majority of laboratories had a laboratory information system (LIS) in place, only half had the capacity to extract data directly from the LIS for the purpose of AMR surveillance; 73.6% (53/72) used restrictive antibiograms. Public microbiology laboratory AMR capacities in Greece require improvement, prioritising interventions for EUCAST guidelines implementation.

## Background

Antimicrobial resistance (AMR) is one of the top 10 global public health threats facing humanity, according to the World Health Organization (WHO) [1]. In 2019, the United States (US) Centers for Disease Control and Prevention (CDC) reported that more than 2.8 million antimicrobial-resistant infections occur in the US each year, while more than 35,000 patients die because of AMR and its consequences [2]. Data from the European Antimicrobial Resistance Surveillance Network (EARS-Net) estimated that in 2020 more than 800,000 infections occurred in the European Union/European Economic Area (EU/EEA) because of antibiotic resistant bacteria, while more than 35,000 people died as a direct consequence of these infections [3]. 

Meanwhile, last-line antibiotics are failing and nosocomial infections are difficult to treat [4,5]. If proactive measures are not implemented, deaths because of AMR could reach 10 million annually by the year 2050, incurring a total global cost of 100 trillion US dollars (EUR 91 trillion) [5]. At the same time, projected trends in the EU/EEA estimate that up to EUR 1.1 billion are expected to be spent yearly between 2015 and 2050 to address AMR. This corresponds to about 1.8 EUR per capita per year on average, while in countries with high AMR rates (Italy, Malta, Luxembourg and Greece), this cost reaches about 4.1–4.8 EUR per capita per year [6].

Antimicrobial resistance in Greece is among the highest across the EU/EEA. The latest report (2021) from the EARS-Net revealed that Greece ranked first in combined resistance of *Klebsiella pneumoniae* to third-generation cephalosporins, fluoroquinolones and aminoglycosides (resistance rate (RR): 67.4%), second in meticillin resistance of *Staphylococcus aureus* (RR: 41.9%) and third in combined resistance of *Acinetobacter baumannii* to fluoroquinolones, aminoglycosides and carbapenems (RR: 91.4%) [7]. Antimicrobial resistance also affects paediatric patients in Greece, as demonstrated in a 2019 study, which reported 100% resistance of *Acinetobacter* species to carbapenems in paediatric and neonatal intensive care units and in haematology/oncology units [8].

Surveillance is the cornerstone for designing and implementing infection prevention and control measures and relies on diagnostic laboratory capacity for prompt and accurate pathogen identification and antimicrobial susceptibility testing (AST) [1]. There are two international organisations that provide the interpretive criteria for in vitro susceptibility data of pathogens: the Clinical and Laboratory Standards Institute (CLSI) in the United States [9] and the European Committee on Antimicrobial Susceptibility Testing (EUCAST) [10]. In 2018, the European Commission enacted legislation promoting the use of EUCAST among EU countries, a measure also adopted by the EARS-Net reporting protocol, which began accepting data only from EUCAST laboratories in 2019 [11,12]. European countries as well as others worldwide have adopted EUCAST for their everyday microbiology practice and patient care, ensuring comparable AMR results between different settings and standardised AMR surveillance processes [13].

In this Perspective, we examine the practices and capacities for pathogen/species identification and AST in the microbiology labs of the public healthcare sector in Greece.

## Current situation in Greece

WHONET-Greece is the national network responsible for AMR surveillance in Greek hospitals. Initiated in 1995, it uses routine AST testing data of clinical samples processed at hospital microbiology labs [14,15]. WHONET-Greece participates in both the EARS-Net and the Global Antimicrobial Surveillance System (GLASS) [14]. 

In Greece, microbiology laboratories were initially aligned to CLSI guidelines, and adoption of EUCAST guidelines has been slow [16]. The variability in AMR surveillance practices means that data contributed to EARS-Net by the WHONET-Greece have decreased in the past 3 years, as data contribution is limited to only EUCAST-aligned laboratories. The continued use of CLSI guidelines in Greece is likely due to the challenging conditions that the public hospital sector and the microbiology laboratories experienced after the financial crisis of 2009. The lack of specialised staff (microbiologists and technicians) predisposes the personnel negatively to any change in practice as it seen as an additional burden. The substantial increase in workload during the COVID-19 pandemic could have been another reason to enhanced resistance to change. The switch to EUCAST guidelines, which would require human resources, time and effort, could seem overwhelming, especially if the added value of such a change is underestimated and only vaguely understood. 

The inconsistency in AMR microbiology laboratory practices in Greece adds to the present AMR surveillance limitations, and likely contributes to the high levels of AMR in the country. This inconsistency, combined with the high rates of healthcare-associated infections and the high antibiotic consumption, especially with regard to broad-spectrum antibiotics, adds to the problem [17-19].

Under this scope, the Greek National Public Health Organisation (NPHO) surveyed hospital microbiology laboratories to document the practices and capacities for pathogen/species identification and AST in the microbiology laboratories within the public healthcare sector in Greece, as well as to address inconsistency problems and relevant gaps, focusing on the country’s adoption of EUCAST methodology and guidelines.

## Design and setting of the survey

The NPHO conducted a survey between 1 February and 1 April 2022, among all microbiology laboratories within public healthcare facilities in Greece, including primary, secondary and tertiary healthcare facilities, as well as university hospitals. Additional details on inclusion criteria, definitions of healthcare facilities, statistical analysis etc. can be found in the Supplementary Material S1. All microbiology laboratories in the country that fulfilled the inclusion criteria received a questionnaire via email. The questionnaire consisted of 13 questions, which covered: (i) the type of AST guidelines used (EUCAST vs CLSI), (ii) the types of laboratory testing methods available, (iii) the existence and interconnection of a laboratory information system (LIS) and (iv) the use of restrictive antibiogram for antimicrobial susceptibility reporting. The English translation of the questionnaire is provided in Supplementary Material S2.

## Survey outcome 

The questionnaire was sent to 98 of total 128 microbiology laboratories in the country that fulfilled the inclusion criteria. The 30 excluded were most frequently located within primary healthcare facilities. Of the 98 laboratories included, 72 (73.5%) responded, including 21 of 25 national public hospitals with a capacity of over 400 beds and all seven national university hospital laboratories.

### Antimicrobial susceptibility testing guidelines in use

EUCAST guidelines were used by 51.4% (37/72) of the laboratories, and CLSI guidelines were used by 48.6% (35/72) of the laboratories. One of the laboratories using CLSI had planned to switch to EUCAST guidelines in the month after survey completion, while none used both methods. Most (76.5%; 64/72) of the EUCAST laboratories had started using EUCAST guidelines only after the relevant EU Commission regulation (see Table).

**Table:** Laboratory capacities with regard to pathogen identification and antimicrobial susceptibility testing according to hospital type and hospital bed capacity, Greece, 2022 (n = 72 laboratories)

Variables		Hospital type				Hospital bed capacity				
		Hospitals, total (n = 72)		University hospitals (n = 7)		> 400 beds (n = 21, including 5 university hospitals)		< 400 beds (n = 51, including 2 university hospitals)		Hospitals > 400 vs < 400 beds
		n	%	n	%	n	%	n	%	p value
Antimicrobial susceptibility testing guidelines	EUCAST	37	51.4	5	71.4	13	61.9	24	47.1	0.519
	CLSI	35	48.6	2	28.6	8	38.1	27	52.9	
EUCAST implementation before European Commission regulation [11]		8^a^	23.5	3^b^	75.0	3^c^	23.1	5	23.8	0.999
Automated instrument use for species identification and antibiotic susceptibility testing^d,e^	Systematic	44	66.7	7	100	13	61.9	31	68.9	0.855
	Selective	22	33.3	0	0	8	38.1	14	31.1	
Kirby–Bauer disk diffusion method use		58	80.6	6	85.7	18	85.7	40	78.4	0.777
If the Kirby–Bauer disk diffusion method is used, availability of an automated plate reader		6	10.3	1	16.7	2	11.1	4	10.0	0.992
Broth microdilution use for colistin susceptibility testing		46	63.9	6	85.7	19	90.5	27	52.9	0.011*
MALDI-TOF mass spectrometry use		7	9.7	4	57.1	5	23.8	2	3.9	0.035*
LIS use in the lab		65	90.3	6	85.7	17	81.0	48	94.1	0.230
Connection of LIS with automated instrument for species identification and antibiotic susceptibility testing		38	58.5	6	100	12	70.6	25	52.1	0.416
Connection of LIS with hospital HIS		30	46.2	3	50.0	4	23.5	26	54.2	0.093
Data extraction directly from LIS for purposes of AMR surveillance^f^		31	47.7	5	83.3	11	64.7	20	41.7	0.263
Restrictive antibiogram use^g^		53	73.6	4	57.1	14	66.7	39	76.5	0.692
Restrictive antibiogram use, frequency^g^	Always	8	11.1	2	28.6	2	9.5	6	11.8	
	Often	18	25.0	0	0	3	14.3	15	29.4	
	Sometimes	7	9.7	1	14.3	1	4.8	6	11.8	
	Seldom	20	27.8	1	14.3	8	38.1	12	24.0	
	Never	19	26.4	3	42.9	7	33.3	12	23.1	

AMR: antimicrobial resistance; CLSI: Clinical and Laboratory Standards Institute; EUCAST: European Committee on Antimicrobial Susceptibility Testing; HIS: health information system; LIS: laboratory information system; MALDI-TOF: Matrix-assisted laser desorption ionisation-time of flight.

^a^ 34/37 of the laboratories answered this question.

^b^ 4/5 of the laboratories answered this question.

^c^ 21/24 of the laboratories answered this question.

^d^ Six laboratories do not have the instrument in place.

^e^ Systematic for all clinical specimens, and selective for specific isolates (like blood isolates) or for complicated cases of infection.

^f^ Laboratory capacity to extract AMR data directly from the LIS for the purposes of AMR surveillance (in Greece, to WHONET Greece, the national database collection AMR data from the hospitals).

^g^ Use of restrictive antibiogram for antimicrobial susceptibility reporting in the hospital level (restrictive reporting of selected antimicrobial susceptibilities).

A chi-squared test was used for the correlation between hospitals > 400 and hospitals < 400 beds. P values below 0.05 indicated with an asterisk were considered significant.

EUCAST implementation was higher in university hospitals (5/7) and large hospitals with over 400 beds (13/21), compared with implementation in smaller hospitals with fewer than 400 beds (24/51). However, these differences were not statistically significant.

### Laboratory testing practice

Two of three (66.7%; 44/66) laboratories used an automated instrument for species identification and AST for all samples: 15 used such an instrument selectively for all blood isolates, four for difficult cases only and two for all the resistant pathogens (Table). The Kirby–Bauer disk diffusion method is used in order to determine the susceptibility of pathogenic bacteria to various antimicrobial compounds, and support clinicians with their decision regarding antimicrobial treatment for their patients. This method was used by 80.6% (58/72) of the microbiology laboratories. However, only 10.3% (n = 6) of those laboratories reported having an automated plate reader in place, which provides rapid and accurate phenotypic AST data for several available antibiotics in a single test. Hospital type did not impact the existence and use of the Kirby–Bauer method (Table).

Both EUCAST and CLSI recommend broth microdilution (BMD) for antimicrobial susceptibility testing of colistin [20]. This recommendation was also adopted by the Greek National Antibiogram Committee (NAC) and an increasing number of hospital laboratories have complied. The broth microdilution method for colistin susceptibility testing was used by 63.9% (46/72) of the laboratories. However, this method was used more frequently in larger hospitals with over 400 beds than in hospitals with < 400 beds (90.5% vs 52.9% respectively, p = 0.011, Table). Determining colistin resistance in an accurate way is considered very important in Greece, since resistance to colistin is often observed by clinicians, but cumulative data with regard to colistin resistance of pathogens in a national level are lacking [14].

Matrix-assisted laser desorption ionisation-time of flight (MALDI-TOF) mass spectrometry (MS) has been recognised as a rapid and accurate diagnostic tool for pathogen identification, distinguishing bacterial and fungal isolates within minutes [21]. MALDI-TOF MS was, however, available only in around one in 10 microbiology laboratories that responded (7/72, 9.7%). Again, this method was available more frequently in large hospitals compared with smaller hospitals (23.8% versus 3.9% respectively, p = 0.035), but was mainly used in university hospitals (Table).

### Laboratory information system: existence and interconnection

Laboratory information systems have been developed to organise laboratory results in patient records, as well as to link data from different laboratories (haematology, biochemistry, microbiology, etc.), laboratories and clinicians. Use of LIS also enables connection between laboratories and hospital records in terms of financial aspects (through interconnection with the hospital information system (HIS)). The LIS is an essential tool to manage the information flow between healthcare providers, patients and laboratories [22].

The majority of the microbiology laboratories that responded to the questionnaire (90.3%) reported having an LIS system in place, and this percentage did not differ between hospital types (Table). The LIS was connected to the automated instrument for species identification and AST for 58.5% (38/65) of the laboratories, while LIS was connected to the hospital HIS in 46.2% (30/65) of the laboratories. Less than half of the laboratories (47.7%) had the capacity to extract AMR data directly from the LIS system for the purposes of the Greek AMR surveillance system (WHONET-Greece) [14].

### Use of restrictive antibiogram for antimicrobial susceptibility reporting

A restrictive antibiogram is a method of selectively reporting antibiotic susceptibility tests depending on the microorganism identified, and it is thought to be an essential part of antimicrobial stewardship programs according to the Infectious Disease Society of America (IDSA), leading to immediate reductions in antimicrobial prescribing and cost [23]. Restrictive antibiograms reduce the number of performed complicated AST diagnostics, helping clinicians treat the majority of the patients before deciding to proceed to specific antimicrobial susceptibility tests in more complicated cases of infection.

Almost three of four (73.6%; 53/72) laboratories used restrictive antibiograms. However, there was a variability in how often this method was used. Only 11.1% (8/72) of the laboratories reported that they always use it, one quarter (25.0%; 18/72) used it often, 27.7% (20/72) used it seldom and 8.3% (6/72) used it selectively (for community specimens, urine cultures, or according to the hospitalisation unit and the hospital pathogen epidemiology). The use of restrictive antibiogram was more likely to be used in laboratories of smaller hospitals with fewer than 400 beds (76.5%; 39/51) than in laboratories of larger hospitals with more than 400 beds (66.7%; 14/21). The choice of antimicrobial susceptibility testing guidelines (EUCAST vs CLSI) did not affect the use of restrictive antibiogram (p = 0.804), regardless of the hospital type (over 400 beds, p = 0.817; university hospitals, p = 0.971; less than 400 beds, p = 0.973).

### Adoption of EUCAST guidelines

In Greece, there has been a gradual adoption of EUCAST guidelines; from less than 10% of laboratories in 2013 [16] to 10–50% of the laboratories in April of 2021 [13]. We found that slightly more than 50% of the participating microbiology laboratories have adopted EUCAST breakpoints, mostly laboratories in university and larger hospitals; confirming the slow process. Our results are comparable to the 50–65% reported recently in Spain [24]. 

Other countries within and outside the EU will be faced with the similar challenges, and possible implications, such as changes in the resistance percentages and consequently the epidemiology of local resistance patterns, are already seen on the horizon [25-28]. However, despite the potential drawbacks, it is important to note that EUCAST standards are grounded in well-supported pharmacodynamic-pharmacokinetic research, the guidelines are easily and freely accessible online, and they provide a convenient mechanism for comparing data across countries that are in the same extended geographic area. Another advantage of using EUCAST guidelines is the fact that CLSI guidelines have an annual cost, which can be an additional burden in under-resourced healthcare systems [27], whether in low-middle income countries (LMIC) or high-income countries (HIC). However, EUCAST uses horse blood for Mueller–Hinton agar (MHA) which can be hard to find in some areas such as South-East Asia [28]. 

For Greece, but also for other countries in the European and Mediterranean area, adopting a single system will be an important step towards standardisation. Using the same system with our surrounding countries means that data will not only be comparable within Greece, since all laboratories will be following the same guidelines, but also with laboratories in other countries with which we collaborate. 

Harmonisation and standardisation are important in the fight against antimicrobial resistance and the achievement of better patient outcomes. By using the same guidelines and methods, data collected and compared across different regions can provide valuable information on the emergence and spread of resistant microorganisms. Clear and consistent guidelines on the selection and use of antimicrobial agents ensure that healthcare professionals use drugs appropriately and only when necessary, helping to preserve their effectiveness for future generations, while at the same time warranting that the standards are applied across different regions and countries.

Finally, according to our results, the use of novel technologies for rapid and accurate pathogen identification, such as MALDI-TOF, is limited in the Greek hospital microbiology laboratories. Laboratory information systems, even though available, lack the interconnection with the species identification system or the HIS as well. Our findings are consistent with a recent European Centre for Disease Prevention and Control (ECDC) report, which suggests that not all countries in the EU/EEA have the necessary laboratory capacity to deliver fully effective public health surveillance, as well as to respond to infection threats. Furthermore, there are several gaps with regard to digital interconnection between peripheral laboratories and public health information systems [29].

## Conclusion

Harmonisation and standardisation are crucial in addressing antimicrobial resistance, and they are also important for the achievement of better patient outcomes. However, the slow process of integrating harmonised methodology in Greece, along with the gaps in LIS, HIS and species identification systems highlights the limited capacity in Greece to automatically extract data from hospital microbiology laboratories, and subsequently link them with national data on AMR and antimicrobial consumption. Consequently, the surveillance and effective management of the AMR silent pandemic on a national but also on a European level could be impacted negatively.

## Supplementary Data

101000 Supplement
