# Supplementary material for: Antimicrobial susceptibility testing and reporting practices of public hospital microbiology laboratories in Greece, 2022: A national observational survey and call for action
Source: Euro Surveill. 2023 Aug 24;28(34):2200766. doi: 10.2807/1560-7917.ES.2023.28.34.2200766 (PMC10451010; doi:10.2807/1560-7917.ES.2023.28.34.2200766)
Supplement: Supplement [file 22-00766_KARAGIANNIDOU_SUPPLEMENT.pdf]

This supplementary material is hosted by Eurosurveillance as supporting information alongside the article "Antimicrobial susceptibility testing and reporting practices of public hospital microbiology laboratories in Greece, 2022: A national observational survey and call for action" on behalf of the authors who remain responsible for the accuracy and appropriateness of the content. The same standards for ethics, copyright, attributions and permissions as for the article apply. Eurosurveillance is not responsible for the maintenance of any links or email addresses provided therein.

SUPPLEMENTARY MATERIAL S1 - The methodology of our study, aimed to investigate Greek Public Microbiology Lab capacities with regard to pathogen identification and antibiotic susceptibility testing.

Study design and setting

A cross-sectional observational study was performed between February 1st and April 1st 2022. The study sample consisted of all the microbiology laboratories within public healthcare facilities in Greece, including primary, secondary and tertiary healthcare facilities, as well as university hospitals.

Inclusion criteria

- Microbiology laboratories with the capacity to perform pathogen identification with specimen cultures and antibiograms for any clinical specimen (not performing solely gram strains)
- Labs within public healthcare facilities in Greece
- Labs that provide contact details including e-mail addresses, not only telephone numbers, to the Greek National Public Health Organization (NPHO).

Definition of healthcare facilities [1]

- Primary: healthcare facilities that provide medical services with regard to basic medical subspecialties: General Medical Practice, Internal Medicine, General Surgery, Pediatrics, Obstetrics/Gynecology, and Microbiology with “basic” diagnostic capacities.
- Secondary: except for primary healthcare services, they additionally provide services within 5-10 medical subspecialties in total (Cardiology, Hematology/Oncology, Nephrology, Gastroenterology, Dermatology, Radiology etc.), including Intensive Care therapy. Secondary hospitals also accept referrals from primary hospitals.
- Tertiary: hospitals that provide specialized medical services, in terms of specialized personnel and technical equipment (Neurosurgery, specialized Intensive Care Units, Transplantation Units, specialized Radiology techniques and microbiology laboratory methods etc.). Those hospitals act as referral centers and have a large population coverage. Tertiary hospitals include university hospitals and they are further subcategorized to hospitals with a capacity of <400 beds and hospitals with the capacity of >400 beds.

Questionnaire

A questionnaire was constructed in Google Forms, and the relevant link was electronically distributed to all microbiology laboratories in the country that fulfilled the inclusion criteria, and specifically to the provided e-mail addresses. The e-mail was accompanied by a letter, by which the president of the Greek NPHO was requesting the laboratory response, providing also instructions on how to answer the questions. The responsible that was requested to answer the questionnaire within the laboratory was the official Director Microbiologist or the

Microbiologist temporarily in head of the lab. A kind reminder to answer the questionnaire was sent one month after the first e-mail was delivered.

The questionnaire consisted of 13 questions (the full version of the questionnaire translated in English is provided in Table 1). The questions covered the type of AST guidelines used (EUCAST versus CLSI), the types of laboratory testing methods available, the existence of a Laboratory Information System (LIS), and relevant reporting practices and capacities.

Ethical approval was not obtained for this study, since this was an attempt of the Greek NPHO to assess the adoption of EUCAST methodology by the public microbiology labs in our country, as well as to address the present gaps with regard to the lab capacities to identify microbial species and perform AST.

Statistical methods

Descriptive statistics are presented as absolute (n) and relative frequencies (%). Results are provided for the total of the microbiology labs, as well as according to hospital type; university hospitals, hospitals with a capacity of >400 beds, and hospitals with a capacity of <400 beds. This categorization is the one used by the Greek Ministry of Health. Correlations of interest were evaluated on a univariate level using the chi-square test of independence (with Fisher's exact method when needed). Statistical significance was set to α=5%.

References

[1] Health Bill 2016 n.d. https://mve.gr/documents/bills2016/health-bill-2016.pdf (accessed March 8, 2023).

SUPPLEMENTARY MATERIAL S2 - The translated questionnaire, constructed to investigate Greek Public Microbiology Lab capacities with regard to pathogen identification and antibiotic susceptibility testing.

1. Which Antimicrobial Susceptibility Testing guidelines do you use in your lab?

a. EUCAST
b. CLSI
c. Other, please specify

2. Which automated instrument do you use in your lab for species identification and antibiotic susceptibility testing? (list with companies provided)

3. The use of the automated instrument for species identification and antibiotic susceptibility testing in your lab is:

a. Systematic, including all the lab routine
b. Selective, according to the specimen type, including however all blood isolates
c. Selective, including all complicated and difficult for identification cases
d. Not systematic, the automated instrument is used only when the consumables are available
e. Other, please specify

4. Do you use the Kirby Bauer disk diffusion method in your lab? (Yes/No)

5. If you use the Kirby Bauer disk diffusion method in your lab, which company is your disk provider? (list with companies provided)

6. If you use the Kirby Bauer disk diffusion method in your lab, do you have a plate reader? (Yes/No)

7. Do you use broth microdilution for colistin susceptibility testing? (Yes/No)

8. Do you use MALDI-TOF mass spectrometry in your lab? (Yes/No)

9. Does your lab have a Laboratory Information System (LIS)? (Yes/No)

10. If your lab has a LIS, is it connected to the automated instrument for species identification and antibiotic susceptibility testing? (Yes/No)

11. If your lab has a LIS, is it connected to the hospital Health Information System (HIS)? (Yes/No)

12. How often do you use restrictive antibiogram for antimicrobial susceptibility reporting in your hospital (restrictive reporting of selected antimicrobial susceptibilities)?

a. Always

b. Often

c. Sometimes

d. Seldom

e. Never

f. other, please specify

13. In your lab do you have the capacity to extract AMR data directly from your LIS system for the purposes of AMR surveillance? (Yes/No)
